# 702 Hydroxycobalamin is An Independent Risk Factor for Acute Kidney Injury.

**DOI:** 10.1093/jbcr/irad045.178

**Published:** 2023-05-15

**Authors:** Stephen Gondek, Jill Streams, Robel Beyene, Claci Ayers, Brad Dennis, Christina Hayhurst, Elizabeth Dale Slater, Blair Summitt, Anne Wagner

**Affiliations:** Vanderbilt University Medical Center, Nashville, Tennessee; Vanderbilt University Medical Center, Nashville, Tennessee; Vanderbilt University Medical Center, Nashville, Tennessee; Vanderbilt University Medical Center, Nashville, Tennessee; Vanderbilt University Medical Center, Nashville, Tennessee; Vanderbilt University Medical Center, Nashville, Tennessee; Vanderbilt University Medical Center, Nashville, Tennessee; Vanderbilt University Medical Center, Nashville, Tennessee; Vanderbilt University Medical Center, Nashville, Tennessee; Vanderbilt University Medical Center, Nashville, Tennessee; Vanderbilt University Medical Center, Nashville, Tennessee; Vanderbilt University Medical Center, Nashville, Tennessee; Vanderbilt University Medical Center, Nashville, Tennessee; Vanderbilt University Medical Center, Nashville, Tennessee; Vanderbilt University Medical Center, Nashville, Tennessee; Vanderbilt University Medical Center, Nashville, Tennessee; Vanderbilt University Medical Center, Nashville, Tennessee; Vanderbilt University Medical Center, Nashville, Tennessee; Vanderbilt University Medical Center, Nashville, Tennessee; Vanderbilt University Medical Center, Nashville, Tennessee; Vanderbilt University Medical Center, Nashville, Tennessee; Vanderbilt University Medical Center, Nashville, Tennessee; Vanderbilt University Medical Center, Nashville, Tennessee; Vanderbilt University Medical Center, Nashville, Tennessee; Vanderbilt University Medical Center, Nashville, Tennessee; Vanderbilt University Medical Center, Nashville, Tennessee; Vanderbilt University Medical Center, Nashville, Tennessee; Vanderbilt University Medical Center, Nashville, Tennessee; Vanderbilt University Medical Center, Nashville, Tennessee; Vanderbilt University Medical Center, Nashville, Tennessee; Vanderbilt University Medical Center, Nashville, Tennessee; Vanderbilt University Medical Center, Nashville, Tennessee; Vanderbilt University Medical Center, Nashville, Tennessee; Vanderbilt University Medical Center, Nashville, Tennessee; Vanderbilt University Medical Center, Nashville, Tennessee; Vanderbilt University Medical Center, Nashville, Tennessee; Vanderbilt University Medical Center, Nashville, Tennessee; Vanderbilt University Medical Center, Nashville, Tennessee; Vanderbilt University Medical Center, Nashville, Tennessee; Vanderbilt University Medical Center, Nashville, Tennessee; Vanderbilt University Medical Center, Nashville, Tennessee; Vanderbilt University Medical Center, Nashville, Tennessee; Vanderbilt University Medical Center, Nashville, Tennessee; Vanderbilt University Medical Center, Nashville, Tennessee; Vanderbilt University Medical Center, Nashville, Tennessee; Vanderbilt University Medical Center, Nashville, Tennessee; Vanderbilt University Medical Center, Nashville, Tennessee; Vanderbilt University Medical Center, Nashville, Tennessee; Vanderbilt University Medical Center, Nashville, Tennessee; Vanderbilt University Medical Center, Nashville, Tennessee; Vanderbilt University Medical Center, Nashville, Tennessee; Vanderbilt University Medical Center, Nashville, Tennessee; Vanderbilt University Medical Center, Nashville, Tennessee; Vanderbilt University Medical Center, Nashville, Tennessee; Vanderbilt University Medical Center, Nashville, Tennessee; Vanderbilt University Medical Center, Nashville, Tennessee; Vanderbilt University Medical Center, Nashville, Tennessee; Vanderbilt University Medical Center, Nashville, Tennessee; Vanderbilt University Medical Center, Nashville, Tennessee; Vanderbilt University Medical Center, Nashville, Tennessee; Vanderbilt University Medical Center, Nashville, Tennessee; Vanderbilt University Medical Center, Nashville, Tennessee; Vanderbilt University Medical Center, Nashville, Tennessee; Vanderbilt University Medical Center, Nashville, Tennessee; Vanderbilt University Medical Center, Nashville, Tennessee; Vanderbilt University Medical Center, Nashville, Tennessee; Vanderbilt University Medical Center, Nashville, Tennessee; Vanderbilt University Medical Center, Nashville, Tennessee; Vanderbilt University Medical Center, Nashville, Tennessee; Vanderbilt University Medical Center, Nashville, Tennessee; Vanderbilt University Medical Center, Nashville, Tennessee; Vanderbilt University Medical Center, Nashville, Tennessee; Vanderbilt University Medical Center, Nashville, Tennessee; Vanderbilt University Medical Center, Nashville, Tennessee; Vanderbilt University Medical Center, Nashville, Tennessee; Vanderbilt University Medical Center, Nashville, Tennessee; Vanderbilt University Medical Center, Nashville, Tennessee; Vanderbilt University Medical Center, Nashville, Tennessee; Vanderbilt University Medical Center, Nashville, Tennessee; Vanderbilt University Medical Center, Nashville, Tennessee; Vanderbilt University Medical Center, Nashville, Tennessee

## Abstract

**Introduction:**

Previous studies have reported a correlation between the use of hydroxocobalamin and acute kidney injury (AKI), but this is often attributed to the severity of their burn injury. In this study, we use a matched control group to correct for severity of injury and assess the relationship between hydroxycobalamin and AKI.

**Methods:**

All patients receiving hydroxycobalamin over a three year period were identified. The patients were matched 1:1 based on TBSA and age to create a control group. Creatinine and change in creatinine every 24 hours for the first 72 hours were recorded and compared, with an increase in creatinine >1.5 being considered an AKI. A retrospective matched cohort study was performed using the student’s T-test for continuous data and a chi squared test for categorical data.

**Results:**

A total of 50 patients receiving hydroxycobalamin were identified and matched with 50 patients who did not receive hydroxycobalamin. The groups were no signficant differences between groups in terms of age, TBSA or baseline creatinine. Patient receiving hydroxycobalamin had a mean increase of creatinine of 0.13, 0.13 and 0.07 at 24, 48, 72 hours respectively versus 0.0, 0.04 and 0.0 in the control group (p = .09, .06, .49). Qualitatively, 15/50 patients receiving hydroxycobalamin developed AKI vs 2/50 in the control group (p < .001 ), with 6/33 minor burns developing AKI with hydroxycobalamin vs 1/33 in the control group (p=.058). Five patients receiving hydroxycobalamin developed AKI lasting greater than 72 hours, two of which had only minor burns.

**Conclusions:**

Hydroxycobalamin is an independent risk factor for the development of acute kidney injury, even in the setting of minor burns and should only be used in patients with high risk for cyanide poisoning. Empiric and protocolized use may result in harm to patients and should be discouraged.

**Applicability of Research to Practice:**

This study demonstrates the risk of AKI with the use of hydroxycobalamin in both major and minor burns, which burn centers should consider when creating protocols regarding potential cyanide poisoning.

